# Metabolic reprogramming in diabetic complications: mechanisms, pathologies, and molecular evidence from multi-organ studies

**DOI:** 10.3389/fimmu.2026.1874585

**Published:** 2026-08-06

**Authors:** Qian Gong, Wei Zhao, Jing Xia, Zhiwei Nie, Ruifan Luo, Lingxiu Li, Changwu Dong, Yujiao Zheng

**Affiliations:** College of Traditional Chinese Medicine, Anhui University of Chinese Medicine, Hefei, China

**Keywords:** diabetic complications, ferroptosis, immunometabolism, metabolic memory, metabolic reprogramming, mitochondrial dysfunction

## Abstract

Metabolic reprogramming is a critical link between systemic metabolic dysregulation and organ-specific, persistent injury in diabetic complications. Previous reviews have largely focused on individual organs or isolated metabolic pathways, leaving unresolved how common diabetic metabolic reprogramming is translated into divergent tissue injury across different organs and cell types. Addressing this gap is important because it connects fragmented pathway-level evidence with tissue-specific disease mechanisms and may help prioritize more precise therapeutic strategies for diabetic complications. This review summarizes alterations in glucose, lipid, and amino acid metabolism, mitochondrial function, immunometabolism, and epigenetic regulation in diabetic kidney disease, diabetic retinopathy, diabetic foot ulcers, diabetic peripheral neuropathy, and diabetic cardiovascular complications. Current evidence indicates that hypoxia-inducible factor 1α (HIF-1α)-driven glycolysis, ferroptosis-associated oxidative stress, mitochondrial dysfunction, dysregulated nutrient sensing, and inflammatory metabolic remodeling are shared across multiple diabetic complications. However, their downstream consequences are highly dependent on tissue-specific microenvironments and resident-cell composition. For example, HIF-1α-related glycolytic remodeling promotes macrophage-driven inflammation and fibrosis in diabetic kidney disease but contributes to Müller-cell-derived VEGF/ANGPTL4 expression and pathological angiogenesis in diabetic retinopathy. Similarly, ferroptosis-associated lipid injury causes endothelial repair failure in diabetic foot ulcers but cardiomyocyte injury and cardiac remodeling in diabetic cardiovascular complications. These examples suggest that local oxygen status, metabolic demand, immune-cell composition, intercellular metabolic crosstalk, and tissue repair capacity reshape shared metabolic programs into organ-specific pathological outcomes, including filtration-barrier injury, vascular leakage, impaired wound healing, neuropathic injury, and cardiac dysfunction. Moreover, hyperglycemia-induced oxidative stress, inflammatory metabolic remodeling, and epigenetic alterations may persist after glycemic improvement and contribute to metabolic memory. By integrating evidence across organs and cell types, this review provides a new perspective for understanding why shared metabolic reprogramming in diabetes produces tissue-specific pathological outcomes. Therapeutic strategies should therefore combine glycemic control with interventions targeting both shared metabolic pathways and organ- or cell-specific pathogenic mechanisms.

## 1 Introduction

Diabetes mellitus is a highly prevalent chronic metabolic disease and a major global public health burden. According to the 11th edition of the International Diabetes Federation Diabetes Atlas, 589 million adults aged 20–79 years were living with diabetes worldwide in 2024, and this number is projected to increase to 853 million by 2050 (1). Chronic diabetic complications, including diabetic kidney disease (DKD), diabetic retinopathy (DR), diabetic foot ulcer (DFU), diabetic peripheral neuropathy (DPN), and cardiovascular complications, are major contributors to disability, organ failure, and reduced quality of life (2, 3). However, traditional injury pathways do not fully explain why tissue damage may persist in some organs even after glycemic control has improved, a phenomenon known as “metabolic memory” (2, 4).

Broadly, metabolic reprogramming refers to the coordinated remodeling of substrate utilization, metabolic pathway activity, and metabolic flux in response to physiological or pathological stimuli, allowing cells to adapt to changes in energy supply, biosynthetic demand, and functional state (5, 6). In diabetes, chronic hyperglycemia, dysregulated lipid metabolism, oxidative stress, and inflammation can jointly disturb cellular metabolic homeostasis (2, 3). These alterations involve not only glucose, lipid, and amino acid metabolism and mitochondrial function, but also immunometabolism and metabolic–epigenetic regulation (4–6).

Chronic hyperglycemia is a major shared upstream driver of tissue injury in diabetes. In the unifying mechanism proposed by Brownlee, intracellular glucose overload increases mitochondrial superoxide production and diverts excess glucose or glycolytic intermediates into the polyol, AGE-formation, diacylglycerol–protein kinase C, and hexosamine pathways (7). More recent studies have begun to incorporate the accumulation of AGEs and abnormal activation of the polyol, hexosamine, and diacylglycerol–protein kinase C pathways into the framework of hyperglycemia-induced metabolic reprogramming in diabetic vascular endothelial injury (8). Therefore, from the perspective of modern metabolic research, these classical hyperglycemia-induced injury pathways can be regarded as components of pathological glucose metabolic reprogramming.

Through distinct but interconnected mechanisms, these pathways promote redox imbalance, activation of pro-inflammatory signaling, endothelial and vascular dysfunction, and abnormal production and modification of extracellular matrix, ultimately leading to cellular dysfunction and tissue injury (2, 7). In addition to abnormal glucose metabolism, diabetes-associated metabolic alterations also involve lipid and amino acid metabolism and mitochondrial function (2, 3), as well as immunometabolism (5) and metabolic–epigenetic regulation (4, 6). Some of these metabolic and epigenetic alterations may persist after glycemic improvement and contribute to the development of diabetic metabolic memory (2, 4).

This review first summarizes the shared mechanisms of metabolic reprogramming in diabetic complications, with emphasis on abnormalities in glucose, lipid, and amino acid metabolism, as well as remodeling of mitochondrial function, redox homeostasis, immunometabolism, and metabolic–epigenetic regulation. On this basis, it further focuses on DKD, DR, DFU, DPN, and diabetic cardiovascular complications, comparing the common features and tissue- or cell-type-specific alterations of metabolic reprogramming across different organs and cell populations, and evaluating the current evidence base and potential clinical significance of these mechanisms.

## 2 Shared mechanisms of metabolic reprogramming in diabetic complications

### 2.1 Glucose metabolic reprogramming

**Figure 1:**
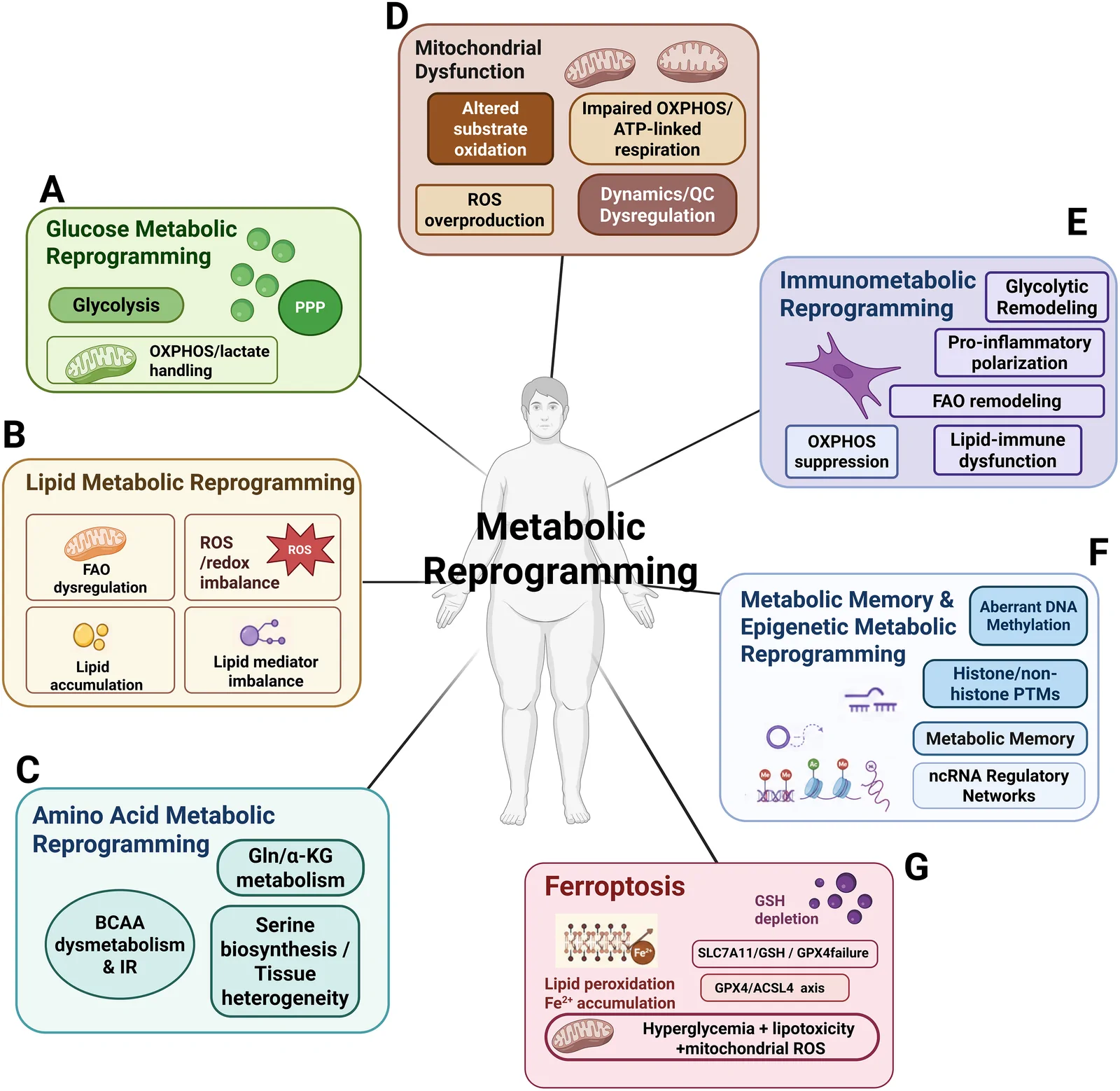
Shared mechanisms of metabolic reprogramming in diabetic complications. **(A)** Glucose metabolic reprogramming; **(B)** lipid metabolic reprogramming; **(C)** amino acid metabolic reprogramming; **(D)** mitochondrial dysfunction; **(E)** immunometabolic reprogramming; **(F)** metabolic memory and epigenetic metabolic reprogramming; and **(G)** ferroptosis. Created with BioRender.com.

Under physiological conditions, glucose metabolism is balanced among glycolysis, the pentose phosphate pathway (PPP), and mitochondrial oxidative phosphorylation. In diabetic complications, this balance is disturbed in a cell- and tissue-dependent manner, with evidence from DKD, DR, and DPN linking altered glycolysis, lactate handling, mitochondrial metabolism, and G6PD/PPP activity to tissue injury and inflammation (9–14) (**Figure 1A**). Enhanced glycolysis is a key component of metabolic reprogramming in diabetic complications. Across DKD, DPN, DCM, and DFU, sustained hyperglycemia is associated with disease- and cell-specific shifts between glycolysis and mitochondrial oxidative metabolism, often accompanied by altered lactate production or handling. These changes contribute to mitochondrial oxidative injury, inflammatory remodeling, and impaired tissue repair (15–18). The Pentose Phosphate Pathway (PPP) provides reduced nicotinamide adenine dinucleotide phosphate (NADPH) and ribose-5-phosphate for redox control and biosynthesis (19–21). In DR, altered G6PD activity has been associated with inflammatory factors, suggesting that PPP-dependent redox balance may influence retinal inflammation (11). Functional variation in transketolase, a non-oxidative PPP enzyme, has also been associated with diabetic polyneuropathy in a population-based study, although this evidence is associative rather than mechanistic (22). In DKD, NADPH consumption induced by aldose reductase (AR) drives the PPP, which in turn activates protein kinase C (PKC), increases prostaglandin production in the glomerulus, and leads to a loss of contractility in mesangial cells (23, 24).

### 2.2 Lipid metabolic reprogramming

Lipid metabolism reprogramming refers to changes in lipid uptake, intracellular lipid storage, lipolysis, fatty acid oxidation, lipid droplet turnover, cholesterol processing, and lipid mediator balance associated with diabetes. Among the diabetic complications, these changes manifest not only as systemic dyslipidemia but also as localized metabolic remodeling in target organs and specific cell types (25, 26) (**Figure 1B**). In DR, hyperglycemia can inhibit AMPK-mediated fatty acid oxidation (FAO), triggering lipid metabolic reprogramming, which manifests as impaired FAO activity and mitochondrial redox imbalance, thereby leading to impaired barrier function of retinal endothelial cells and vascular leakage (27). In DKD, hyperglycemia enhances dicarboxylic acid oxidation in renal peroxisomes, leading to increased succinate production. Accumulated succinate inhibits mitochondrial FAO, resulting in impaired fatty acid oxidation, lipid accumulation, excessive ROS production, and glomerular injury (28).

### 2.3 Amino acid metabolic reprogramming

Amino acid metabolism reprogramming in diabetes involves altered branched-chain amino acid (BCAA) catabolism, glutamine/α-ketoglutarate (α-KG) metabolism, and serine biosynthesis (29–33). These alterations are associated with insulin resistance, mitochondrial dysfunction, redox imbalance, and impaired tissue repair (**Figure 1C**). In DKD, impaired BCAA catabolism and disruption of BCAA homeostasis promote inflammatory and fibrotic injury (34). In diabetic myocardium, BCAA accumulation and broader amino acid and lipid metabolic reprogramming have also been observed (35). Additionally, in DR, the direction of BCAA catabolism is altered, and abnormalities in its metabolic flux are closely associated with the inflammatory response in the retina (36). These findings indicate that amino acid metabolic reprogramming—particularly an imbalance in branched-chain amino acid homeostasis—is a common metabolic abnormality in various diabetic complications.

### 2.4 Mitochondrial metabolic reprogramming

Mitochondrial metabolic reprogramming is a major component of metabolic reprogramming in diabetic complications. It includes altered substrate oxidation, impaired oxidative phosphorylation, reduced ATP-linked respiration, excessive reactive oxygen species production, dysregulated mitochondrial dynamics, and defective mitophagy (37, 38) (**Figure 1D**). In DKD and DCM, persistent hyperglycemia and metabolic disturbances induce mitochondrial metabolic reprogramming that may involve overlapping changes in substrate utilization, glycolysis, mitochondrial redox homeostasis, ATP-linked respiration, mitochondrial dynamics, and mitophagy. These alterations impair tissue energy metabolism, increase oxidative stress, and compromise cellular repair, thereby contributing to the progression of diabetic complications (39–41).

### 2.5 Immunometabolic remodeling and immune cell polarization

Immune metabolic reprogramming refers to the systematic adjustment of metabolic pathways in immune cells in response to pathological stimuli, including enhanced glycolysis, alterations in oxidative phosphorylation, and the remodeling of fatty acid oxidation. These metabolic changes directly drive the polarization of immune cells toward pro-inflammatory or anti-inflammatory phenotypes (42–44). In complications such as DKD, DR, and DFU, the pathological microenvironment shaped by hyperglycemia and dyslipidemia can drive metabolic reprogramming of immune cells, primarily manifested as enhanced glycolytic flux and suppressed oxidative phosphorylation. This metabolic reprogramming further promotes the polarization of immune cells toward a pro-inflammatory phenotype, thereby contributing to the progression of various complications (45–49).

### 2.6 Metabolic memory and epigenetic metabolic reprogramming

Metabolic memory refers to the persistent effect of prior hyperglycemic exposure on later complication risk, even after subsequent improvement in glycemic control. Mechanistically, hyperglycemia-driven metabolic stress may leave durable epigenetic and transcriptional imprints that sustain inflammatory or reparative dysfunction after glucose normalization (50). Evidence suggests that metabolic memory is mediated by persistent epigenetic changes induced by transient or early metabolic stress (51–53). Among the complications of diabetes, early metabolic damage reprograms chromatin through three interrelated mechanisms—resetting of DNA methylation, redistribution of histone modifications, and remodeling of non-coding RNA networks—which act synergistically to alter chromatin accessibility, transforming transient hyperglycemic signals into long-term pathogenic transcriptional output. These established epigenetic marks persist even after blood glucose levels return to normal, causing cells to maintain abnormal expression of pro-inflammatory and pro-fibrotic genes (52, 54–56). This common mechanism explains why, even after blood glucose levels are brought under control, renal inflammation and fibrosis in DKD, retinal microvascular damage in DR, and neuroinflammation and axonal damage in DPN continue to progress, forming the pathogenic basis of the “metabolic memory” of diabetic complications.

### 2.7 Ferroptosis

Biochemically, ferroptosis is characterized by the accumulation of oxidation-prone polyunsaturated phospholipids, redox-active iron, glutathione depletion, and impaired detoxification of lipid peroxides through the SLC7A11/GSH/GPX4 antioxidant system (57, 58). Under diabetic conditions, hyperglycemia, lipotoxicity, and mitochondrial reactive oxygen species may converge to promote phospholipid peroxidation and weaken GPX4-dependent antioxidant defense, thereby altering cellular susceptibility to ferroptosis. In β-cell models exposed to glucolipotoxic stress, ferroptotic injury has been associated with dysregulation of the GPX4/ACSL4 axis, linking lipid peroxidation to β-cell dysfunction and injury (59).

## 3 Diabetic kidney disease and metabolic reprogramming

**Figure 2:**
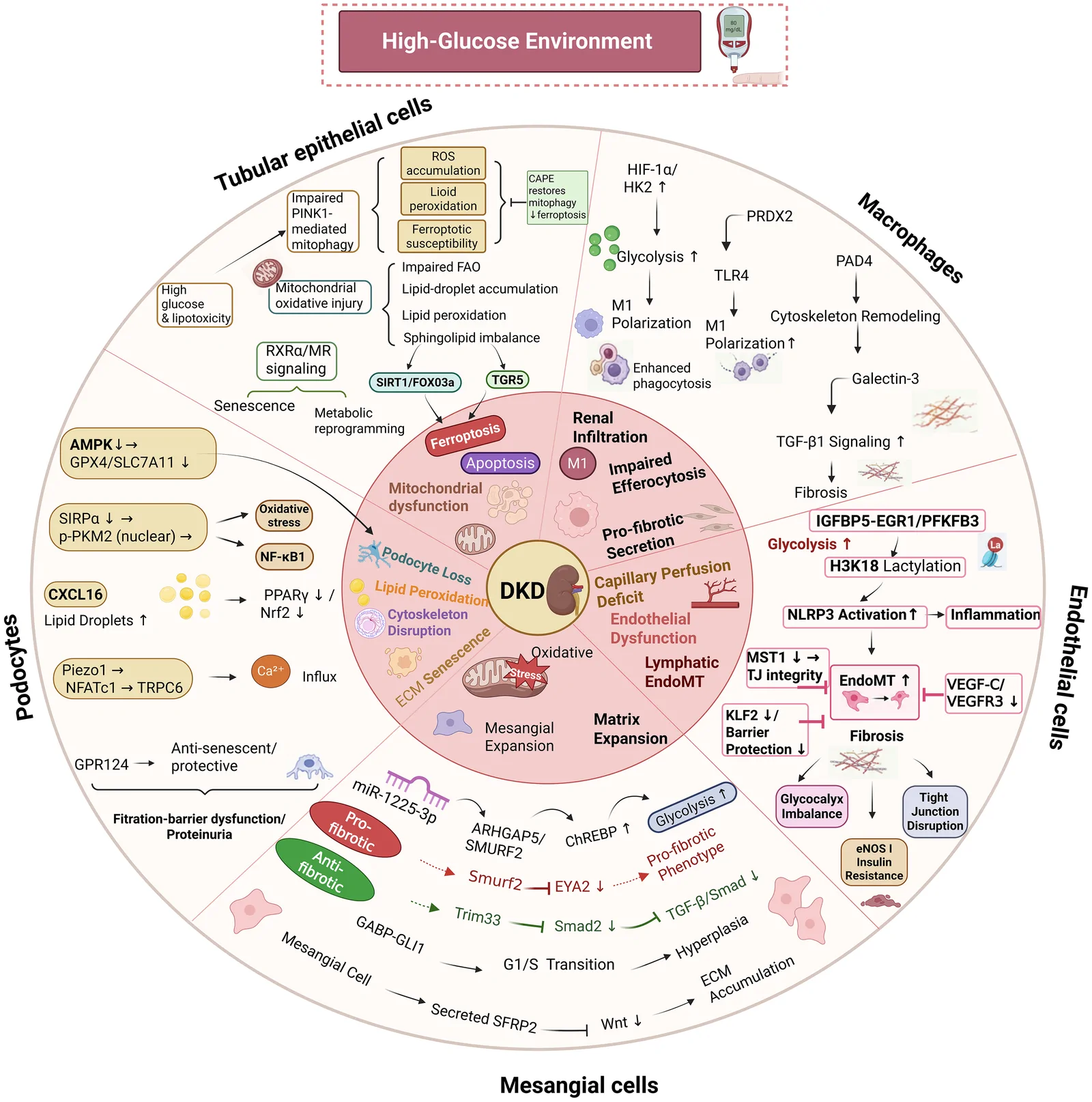
Cell-type-specific metabolic reprogramming in diabetic kidney disease. In the high-glucose diabetic kidney, tubular epithelial cells, podocytes, mesangial cells, endothelial cells, and macrophages undergo distinct metabolic and immunometabolic remodeling. These changes involve mitochondrial dysfunction, impaired mitophagy, lipid peroxidation/ferroptosis, glycolytic inflammation, defective efferocytosis, endothelial dysfunction, matrix expansion, and fibrosis. Created with BioRender.com.

Building on the common mechanisms described above, metabolic reprogramming in DKD shows clear cell-type specificity. Different renal cells do not undergo uniform metabolic injury; instead, they convert diabetic metabolic stress into distinct pathological outcomes, including tubulointerstitial injury, filtration-barrier disruption, mesangial expansion, microvascular dysfunction, and immune-fibrotic amplification (60, 61) (**Table 1**, **Figure 2**).

**Table 1:** Representative organ- and cell-type-specific metabolic reprogramming mechanisms in diabetic complications.

Diabetic complication	Tissue/cell type	Metabolic reprogramming mechanism	Key target/pathway	Evidence models	References
DKD	Tubular epithelial cells	Mitophagy/mitochondrial quality control↓; lipid metabolic imbalance; ferroptosis↑; senescence↑	PINK1/mitophagy/ferroptosis/CAPE; FAO; RXRα/MR	STZ/HFD-induced DBA/2J mice; HG-treated tubular epithelial cells; db/db mice; HG-treated HK-2 cells; diabetic kidney and renal tubular epithelial-cell models	(62–64)
	Podocytes	Energy sensing↓; antioxidant defense↓; ferroptosis↑; oxidative inflammation↑	AMPK/GPX4/SLC7A11; SIRPα/PKM2/NF-κB	Experimental DKD and podocyte models; DKD podocyte models	(68, 69)
	Mesangial cells	Oxidative stress↑; fibrosis/matrix expansion↑; TGF-β/Smad signaling↑	miR-1225-3p/ARHGAP5/SMURF2/ChREBP; Trim33/Smad2	DKD animal/cell models; HG-treated mesangial cells; mesangial-cell fibrosis models	(74, 76)
	Glomerular endothelial cells	Glycolysis/lactylation↑; inflammation/fibrosis↑; EndoMT↑; barrier dysfunction↑	IGFBP5/EGR1/PFKFB3; H3K18 lactylation/NLRP3/EndoMT	DKD mouse and endothelial-cell models; diabetic nephropathy endothelial/renal fibrosis models	(79, 80)
	Lymphatic endothelial cells	Lymphangiogenesis↑; lymphatic EndoMT↑; renal fibrosis↑	VEGF-C/VEGFR3; empagliflozin	Diabetic kidney disease mouse models	(83)
	Macrophages	Glycolysis↑; macrophage recruitment↑; tubulointerstitial inflammation↑; fibrosis↑	HIF-1α/HK2; PAD4/p65/Cmklr1; Galectin-3/TGF-β1	Macrophage-related DKD models; DKD patient samples; diabetic animal models; macrophage-specific interventions; DKD macrophage/renal fibrosis models	(84, 87, 89)
DR	Retinal endothelial cells	Mitochondrial dysfunction↑; glycolysis/angiogenesis dysregulation↑; barrier leakage↑	Endothelial mitochondrial programs; tsRNA-1599; TG2/AMPK/GAPDH	Human PDR omics/subtype analysis; ocular endothelial-cell models; diabetic retina/endothelial models	(90, 93, 94)
	Retinal pericytes	Glucose-flux diversion↑; mitochondrial bioenergetics↓; redox failure/apoptosis↑	Polyol pathway; mitochondrial morphology/metabolism; TXNIP	High-glucose retinal pericyte and endothelial-cell models; high-glucose retinal pericytes	(102, 104, 105)
	Müller cells	Ferroptosis/redox-lipid stress↑; DAMP-related inflammation↑; neuroinflammation↑	Müller-cell ferroptosis/DAMP signaling	Hyperglycemic Müller-cell/DR-related models	(109)
	Microglia	Microglial state remodeling↑; mtDNA leakage↑; STING/innate immune activation↑	Retinal microglial subtypes; STING	Single-cell RNA sequencing of early DR retinas; HG/hypoxia-treated microglia; experimental DR models	(111, 112)
	Macrophages	Epitranscriptomic vascular protection↓; lipid-mediator remodeling↑; vascular leakage↑	FTO/m6A/YTHDF2/FGF2/PI3K-AKT; cSPECC1/eIF4A3/GPX2/12-HETE	HG macrophages; diabetic mouse retinas; HG THP-1-derived macrophages; diabetic mice	(117, 118)
	Hyalocytes/macrophage-lineage cells	Vitreous inflammatory/angiogenic/heme-catabolic remodeling↑	IL6/ANGPT2/hemoglobin catabolism/erythrophagocytosis	Human PDR vitreous hyalocyte evidence	(92)
DFU	Endothelial cells	Mitochondrial bioenergetics↓; angiogenesis↓; ferroptosis/iron-redox stress↑	PPAR/SHH/OXPHOS; NETs/PI3K-AKT/ferroptosis; iron/redox balance	Human DFU tissues; diabetic wound models; endothelial-cell models; MSC-EV intervention; multifunctional hydrogel treatment	(124–126)
	Fibroblasts	Glycolytic adaptation↓; mitophagy↓; senescence↑; ferritinophagy/ferroptosis imbalance	PDK4/glycolysis/YAP-JNK; PINK1/Parkin; NCOA4/ferritinophagy/ferroptosis	HG-induced senescent fibroblasts; diabetic wound models; HG-induced dermal fibroblast senescence; DFU rat wound models; diabetic wound tissue; senescent fibroblast models	(131, 134, 135)
	Keratinocytes/fibroblasts	Vesicle-mediated autophagy disruption↑; repair-cell dysfunction↑	Keratinocyte-derived sEVs/fibroblast autophagy	Diabetic wound and keratinocyte-fibroblast crosstalk models	(136)
	Fibroblasts/macrophages	Exosomal miRNA-mediated macrophage autophagy↓	Fibroblast exosomal miR-93-5p/ATG16L1	DFU fibroblast exosome and macrophage models	(137)
	Macrophages/fibroblasts	Persistent inflammatory crosstalk↑ under HG/LPS stress	LPS/HG macrophage-fibroblast crosstalk	LPS-infected and hyperglycemic diabetic wound models	(139)
	Macrophages	Mitophagy↓; innate immune activation↑; repair-associated polarization↓; M2-like repair function↓	AhR/mtDNA/cGAS-STING-NLRP3; IL-27/IL-27Rα/STAT3; CD68/CD163	Diabetic wound models; FICZ hydrogel intervention; human DFU tissue	(140–142)
DPN	Schwann cells	Bioenergetics↓; ROS↑; maladaptive peripheral ketogenesis↑	Pemafibrate/FABP; CB1R/Hmgcs2	HG mouse Schwann-cell models; diabetic neuropathy models; Schwann-cell evidence	(143, 145)
	DRG sensory neurons	Ca2+-mitochondrial oxidative injury↑; trophic signaling↓; neuronal apoptosis↑	TRPV4; NGF/TrkA	Diabetic neuropathic pain mouse models; resveratrol intervention; DPN animal/cell models; plantamajoside intervention	(146, 147)
	Satellite glial cells	Sphingolipid remodeling↑; neuron-glia dysfunction↑; glial activation↑	GalCer metabolism; NR2A/Wnt/TLR2	Painful DPN SGC-neuron models; diabetic mouse DRG/SGC models	(148, 150)
	Mast cells	Nutrient/inflammatory activation↑; degranulation↑	GLUT3/ERK/mTOR	Diabetic peripheral neuropathy mouse models; mast-cell-deficient models	(144)
	Macrophages	Neuroinflammatory macrophage infiltration↑; NF-κB/p38 MAPK activation↑	MANF/NF-κB/p38 MAPK	DRG and sciatic nerve in DPN models	(151)
Diabetic cardiovascular complications	Cardiomyocytes	FAO dependence↑; substrate flexibility↓; ferroptosis↑; retinol metabolism disorder↑	DECR1/PDK4/HDAC3/HADHA; RDH10/CD36/GPX4/FSP1	Diabetic cardiomyopathy mouse models; male diabetic cardiomyopathy mouse models	(158–162)
	Vascular endothelial cells	Glycolysis↑; redox/NO signaling dysfunction↑; oxidative inflammation↑	PFKFB3/eNOS/NOX; KLF11/TXNIP	Human and rodent obesity/endothelial dysfunction models; diabetic atherosclerosis models	(164, 165)
	VSMCs	Metabolic-phenotypic transformation↑; contractile phenotype↓; foam-cell/macrophage-like transition↑	KDM5B/DDIT3/HNMT/CYP27A1; E2F5/Wnt/β-catenin; AGE/RAGE/CD68/ACTA2	Human diabetic arterial spatial multi-omics; diabetic atherosclerosis VSMC models; AGE-treated VSMC models	(166–168)
	Macrophages/hematopoietic immune cells	Succinate/lactate-driven inflammatory signaling↑; macrophage senescence↑; plaque inflammation↑	miR-369-3p/succinate/GPR91; GPR132/Src; NLRP3/AIM2	Diabetes-associated atherosclerosis models; patient PBMC associations; diabetic atherosclerosis macrophage/plaque models; diabetes-accelerated atherosclerosis mouse models	(169–171)

↑ indicates activation/upregulation/accumulation/enhancement; ↓ indicates inhibition/downregulation/depletion/reduction. Listed pathways are representative rather than exhaustive.

### 3.1 Tubular epithelial cells

In tubular epithelial cells, DKD-related metabolic injury is mainly characterized by the coupling of mitochondrial quality-control failure, lipid metabolic disorder, and ferroptosis. Because these cells rely heavily on mitochondrial energy metabolism, impaired mitophagy under hyperglycemic and lipotoxic conditions promotes ROS accumulation, lipid peroxidation, and ferroptotic susceptibility. In STZ/HFD-induced DBA/2J mice and high-glucose-treated tubular epithelial cells, defective PINK1-mediated mitophagy aggravates ferroptosis, whereas CAPE restores mitophagy and reduces ferroptotic injury, identifying the mitophagy–ferroptosis axis as a key mechanism of tubular damage (62). Consistently, mitochondrial oxidative injury in db/db mice and high-glucose-treated HK-2 cells reshapes tubular lipid metabolism, leading to impaired fatty acid oxidation, lipid-droplet accumulation, lipid peroxidation, and sphingolipid imbalance (63). Additional studies show that RXRα/MR signaling promotes tubular senescence and metabolic reprogramming, while TGR5- and SIRT1/FOXO3a-related pathways regulate ferroptosis resistance (64–66). Together, these findings indicate that tubular injury in DKD results from impaired mitochondrial quality control, lipid metabolic imbalance, defective redox defense, and cellular senescence (67).

### 3.2 Podocytes

Unlike tubular epithelial cells, where lipotoxicity and ferroptosis predominate, podocyte metabolic injury directly compromises the glomerular filtration barrier. Podocytes depend on foot-process integrity, slit-diaphragm stability, and antioxidant defense; therefore, lipid peroxidation, glucose metabolic disturbance, and mitochondrial stress readily lead to proteinuria and barrier dysfunction. In experimental DKD, impaired AMPK signaling is associated with reduced GPX4 and SLC7A11 expression, increased lipid peroxidation, mitochondrial dysfunction, and podocyte ferroptosis, suggesting that the AMPK–GPX4/SLC7A11 axis links energy sensing, antioxidant defense, and filtration-barrier integrity (68). In podocyte models of DKD, reduced SIRPα promotes PKM2 nuclear translocation, enhancing oxidative stress and NF-κB-mediated inflammation, indicating that altered localization of a glycolytic enzyme can amplify podocyte injury (69). Other studies further show that CXCL16-related lipid accumulation, PIEZO1-mediated Ca^2+^ influx, NFATc1/TRPC6 signaling, and GPR124-related anti-senescent effects contribute to podocyte dysfunction (70–73). Thus, podocyte injury in DKD reflects the combined effects of metabolic stress, mechano-calcium signaling, and senescence, ultimately impairing filtration-barrier integrity.

### 3.3 Mesangial cells

In mesangial cells, DKD-related metabolic reprogramming is mainly reflected by persistent activation of proliferative and fibrotic programs. Unlike tubular epithelial cells and podocytes, which primarily undergo cell injury or barrier dysfunction, mesangial cells tend to convert hyperglycemia, oxidative stress, and abnormal transcriptional regulation into cell proliferation, extracellular matrix deposition, and mesangial expansion. In DKD animal models and high-glucose-treated mesangial cells, miR-1225-3p suppresses ARHGAP5 and affects SMURF2-mediated ChREBP ubiquitination, thereby promoting oxidative stress and fibrosis. This suggests that abnormal ChREBP stabilization links hyperglycemic signaling to mesangial matrix expansion (74). In addition, the Smurf2/EYA2, Trim33/Smad2, and GABP/GLI1 axes regulate ubiquitination, TGF-β/Smad fibrotic signaling, and cell-cycle-related transcription, respectively (75–77). These findings indicate that hyperglycemic metabolic stress drives mesangial proliferation, matrix accumulation, and glomerulosclerosis through transcriptional and proteostatic mechanisms.

### 3.4 Endothelial cells

In DKD, the diabetic metabolic milieu induces glomerular endothelial inflammation and filtration-barrier dysfunction (60). In addition, IGFBP5/TGF-β1-mediated crosstalk between endothelial and tubular epithelial cells may promote renal interstitial fibrosis (78). Multi-omics analyses of human kidney biopsies and insulin-resistant renal cell models show cell-type-specific endothelial responses to insulin resistance and the diabetic renal environment, providing a systematic basis for understanding endothelial injury in DKD (60). Mechanistically, IGFBP5 promotes endothelial glycolysis, lactate production, extracellular acidification, and renal inflammation through the EGR1/PFKFB3 axis; IGFBP5 deficiency or PFKFB3 mutation attenuates inflammatory injury, suggesting that this pathway converts metabolic stress into endothelial inflammation (79). Further studies show that IGFBP5-driven glycolysis promotes H3K18 lactylation, NLRP3 inflammasome activation, endothelial-to-mesenchymal transition, and renal fibrosis, indicating that the glycolysis–lactylation axis links metabolic reprogramming to inflammatory and fibrotic gene programs (80). Beyond this axis, MST1 protects the filtration barrier by restoring endothelial tight junctions and inhibiting YAP1/TEAD signaling, whereas KLF2 maintains glomerular endothelial homeostasis and slows DKD progression (81, 82). In lymphatic endothelial cells, empagliflozin reduces lymphangiogenesis and lymphatic endothelial-to-mesenchymal transition by inhibiting VEGF-C/VEGFR3 signaling, thereby alleviating renal interstitial fibrosis (83).

### 3.5 Macrophages

In DKD, macrophages serve as a key immunometabolic link between metabolic stress, inflammation, and fibrosis. In macrophage-related DKD models, HIF-1α/HK2-mediated glycolysis promotes classical macrophage activation and inflammation. Extracellular vesicles from injured tubular epithelial cells further stabilize HIF-1α and enhance macrophage glycolysis, indicating that glycolytic reprogramming not only shapes the pro-inflammatory macrophage phenotype but also mediates tubular–macrophage metabolic crosstalk (84, 85). In addition, PRDX2 upregulation in stressed tubular epithelial cells promotes classical macrophage activation through TLR4, forming an oxidative stress–inflammation feedback loop (86). At the level of immune-cell recruitment, genetic association studies, DKD patient samples, diabetic animal models, and macrophage-specific interventions show that PAD4 is increased in the tubulointerstitium of DKD kidneys. Macrophage PAD4 deletion or PAD4 inhibition with GSK484 reduces macrophage infiltration and tubulointerstitial injury by blocking the PAD4/p65/Cmklr1 axis (87). Single-cell and bulk RNA sequencing further reveal altered macrophage subsets and efferocytosis-related programs in DKD, which correlate with proteinuria and renal function (88). Mechanistic studies also show that macrophage-derived galectin-3 enhances TGF-β1 signaling and promotes renal fibrosis (89). Overall, impaired macrophage metabolism, defective clearance programs, and pro-fibrotic secretion jointly sustain chronic inflammation and fibrosis in DKD.

## 4 Diabetic retinopathy and metabolic reprogramming

**Figure 3:**
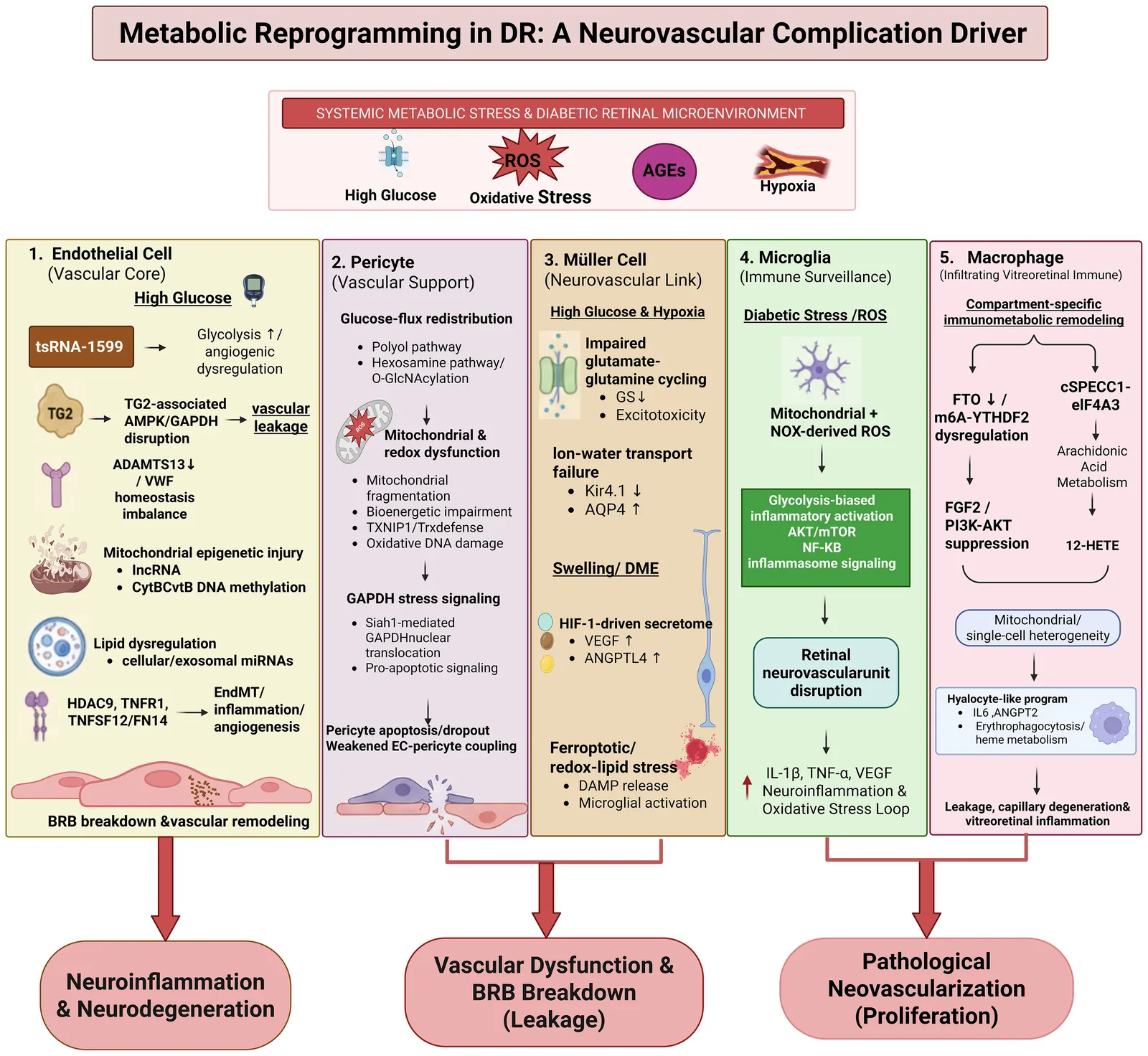
Cell-type-specific metabolic reprogramming in diabetic retinopathy. Systemic metabolic stress, including high glucose, oxidative stress, AGEs, and hypoxia, reprograms retinal endothelial cells, pericytes, Müller cells, microglia, and macrophage-lineage cells. These vascular, glial, and immune alterations drive blood–retinal barrier breakdown, neuroinflammation, neurodegeneration, vascular dysfunction, and pathological neovascularization. Created with BioRender.com.

During DR, hyperglycemia, oxidative stress, hypoxia, and inflammation drive tissue-specific metabolic reprogramming across retinal vascular, glial, and immune cells, contributing to blood–retinal barrier disruption, neuroinflammation, microvascular dysfunction, and pathological angiogenesis (90–92) (**Figure 3**).

### 4.1 Endothelial cells

Within the retinal microvascular compartment, endothelial cells undergo tissue-specific metabolic reprogramming that compromises blood–retinal barrier integrity and promotes inflammatory and angiogenic remodeling. This process first involves glycolytic and angiogenic dysregulation, as tsRNA-1599 upregulation sustains HK2-dependent glycolytic activity and promotes pathological endothelial angiogenesis, whereas its silencing reduces glycolytic capacity and ocular neovascularization (93). Barrier failure is further linked to metabolic control of vascular permeability and hemostatic homeostasis: transglutaminase 2-associated disruption of the AMPK/GAPDH axis is associated with microvascular leakage, whereas ADAMTS13 protects endothelial function by reducing inflammation and restoring VWF-related vascular homeostasis (94, 95). Mitochondrial genome-associated epigenetic remodeling provides another layer of metabolic injury, as altered mitochondrial lncRNA CytB and CytB DNA methylation are associated with mitochondrial instability in diabetic retinal models, while human proliferative diabetic retinopathy omics studies confirm endothelial mitochondrial dysfunction and molecular heterogeneity (90, 96, 97). In parallel, lipid-associated endothelial injury is reflected by altered cellular and exosomal miRNAs, suggesting disturbed lipid handling and endothelial intercellular communication (98). Finally, HDAC9-mediated endothelial–mesenchymal transition, TNFR1-dependent inflammatory signaling, and TNFSF12/FN14-mediated angiogenesis further amplify endothelial dysfunction and vascular remodeling (99–101). Overall, retinal endothelial metabolic reprogramming in DR integrates glycolytic activation, mitochondrial epigenetic injury, lipid dysregulation, inflammation, and angiogenic signaling, ultimately driving blood–retinal barrier breakdown and pathological neovascular remodeling.

### 4.2 Pericytes

Retinal pericytes show a cell-type-specific metabolic vulnerability in diabetic retinopathy because they are critical for capillary stability, endothelial support, and blood–retinal barrier maintenance. Under diabetic stress, excess glucose is diverted from normal energy metabolism into injury-related metabolic pathways. Activation of the polyol pathway and increased hexosamine-pathway flux promote osmotic stress, oxidative stress, and O-GlcNAc-dependent protein modification, thereby linking glucose overload to pericyte loss and loss of vascular support (102, 103). This glucose-flux remodeling is accompanied by mitochondrial and redox dysfunction. High glucose induces mitochondrial fragmentation and bioenergetic impairment, while TXNIP upregulation weakens thioredoxin-dependent antioxidant defense, increases oxidative DNA damage, and promotes pericyte loss (104, 105). These mechanisms indicate that diabetic pericyte injury reflects failure of mitochondrial energy homeostasis and redox buffering rather than isolated apoptotic signaling. In addition, high glucose alters the stress-related function of glycolytic enzymes, as GAPDH nuclear translocation through Siah1 converts a metabolic enzyme into a pro-apoptotic signal (106). Together, these findings suggest that pericyte loss in DR is driven by coordinated glucose-flux redistribution, mitochondrial dysfunction, redox failure, nutrient-sensitive protein modification, and glycolytic enzyme stress signaling. This metabolic remodeling is tissue-relevant because it directly weakens endothelial–pericyte coupling and promotes blood–retinal barrier leakage and retinal microvascular degeneration.

### 4.3 Müller cells

Within the retinal glial compartment, Müller cells exhibit a tissue-specific metabolic vulnerability because they maintain neurotransmitter recycling, ion–water balance, and neurovascular support. In DR, this vulnerability is mainly reflected by disruption of glutamate metabolism, osmotic homeostasis, hypoxia-driven angiogenic signaling, and redox-lipid stress. Müller-cell dysfunction impairs glutamate clearance through reduced glutamine synthetase activity, limiting glutamate-to-glutamine conversion and promoting excitotoxic neuronal injury; concomitant Kir4.1 downregulation and AQP4 upregulation disturb potassium and water transport, contributing to Müller-cell swelling and diabetic macular edema (107). This represents a Müller-cell-specific form of metabolic remodeling because neurotransmitter detoxification and ion–water regulation are core homeostatic functions of retinal Müller glia. Under hypoxic metabolic stress, Müller cells also shift toward a pro-angiogenic secretory phenotype: HIF-1-dependent induction of VEGF and ANGPTL4 increases endothelial permeability and pathological angiogenesis, linking oxygen-metabolic stress to neurovascular injury (108). Redox-lipid injury provides an additional amplification mechanism. Although the core ferroptosis machinery belongs to the common diabetic stress response, Müller-cell ferroptotic injury is tissue-relevant because it promotes DAMP release, microglial activation, and increased inflammatory mediator expression, thereby amplifying retinal neuroinflammation and blood–retinal barrier disruption (109). At the tissue level, alanine–glutamine treatment modulates glutamine metabolism, glycolytic enzymes, inflammatory markers, and VEGF expression in STZ-induced diabetic retina, but because these data were mainly obtained from whole-retina samples, they should be interpreted as supportive retinal-level evidence rather than direct Müller-cell-specific proof (110). Together, these findings suggest that Müller-cell metabolic remodeling in DR is defined by impaired glutamate–glutamine cycling, ion–water transport failure, hypoxia-induced angiogenic signaling, and redox-lipid inflammatory amplification, which jointly disrupt retinal neurovascular homeostasis.

### 4.4 Microglia

Within the retinal immune compartment, diabetic stress promotes microglia-associated immunometabolic remodeling, characterized by redox imbalance, mitochondrial stress, inflammatory activation, and cell-state-specific microglial responses. These changes contribute to retinal inflammation, neurovascular dysfunction, and blood–retinal barrier impairment. Vargas-Soria et al. summarized diabetes-associated alterations in microglial physiology across *in vitro*, preclinical, and clinical studies, emphasizing that microglial activation states vary by model, tissue context, and disease stage (91). Recent single-cell transcriptomic evidence further supports the tissue- and cell-state-specific nature of microglial remodeling in DR. Wang et al. showed that retinal microglia displayed the most prominent transcriptional changes in early DR and identified distinct microglial subtypes within the diabetic retinal microenvironment (111). Mechanistically, Li et al. demonstrated that high glucose or hypoxic stress induces mitochondrial dysfunction and mtDNA leakage in microglia, thereby activating STING-dependent innate immune signaling and aggravating retinal neurovascular unit injury in experimental DR models (112). Activated microglia disrupt the retinal neurovascular unit and contribute to early vascular dysfunction and abnormal blood flow (113). In preclinical models, parthenolide suppresses NF-κB signaling and attenuates microglia-associated Müller-cell gliosis and inflammation (114), whereas IL-4 modulates microglial responses, promotes pericyte survival, and reduces endothelial permeability (115).

### 4.5 Macrophages

Within the retinal immune and vitreoretinal compartments, macrophage-lineage cells show compartment-specific immunometabolic remodeling that links diabetic stress to vascular leakage, capillary degeneration, and vitreoretinal inflammation (116). Beyond the common inflammatory polarization program, one mechanism involves epitranscriptomic control of metabolic and vascular homeostasis. In high-glucose macrophages and diabetic mouse retinas, FTO downregulation disrupts m6A/YTHDF2-dependent regulation and reduces FGF2/PI3K–AKT signaling, thereby weakening macrophage-associated vascular protection and promoting retinal microvascular dysfunction (117). A more direct metabolic mechanism is lipid-mediator reprogramming. In high-glucose THP-1-derived macrophages and diabetic mice, cSPECC1 stabilizes GPX2 mRNA through eIF4A3, reshapes arachidonic acid metabolism, and increases 12-HETE-dependent macrophage–endothelial crosstalk, linking macrophage lipid metabolism to vascular leakage and acellular capillary formation (118). Human transcriptomic and single-cell datasets further identify macrophage- and mitochondria-related signatures in DR, supporting immune-metabolic heterogeneity, although these findings remain mainly associative (119, 120). In the vitreoretinal compartment, hyalocytes exhibit a distinct macrophage-like remodeling program. Increased IL6 and ANGPT2 expression indicates inflammatory and proangiogenic activation, whereas hemoglobin-catabolism-related transcripts and erythrophagocytic activity suggest adaptation to vitreous hemorrhage and local heme metabolism (92). Together, these findings indicate that macrophage-lineage remodeling in DR is not limited to generic M1-like inflammation, but includes epitranscriptomic regulation, lipid-mediator metabolism, mitochondrial-associated immune heterogeneity, and vitreous-specific erythrophagocytic/heme-catabolic remodeling.

## 5 Diabetic foot ulcers and metabolic reprogramming

**Figure 4:**
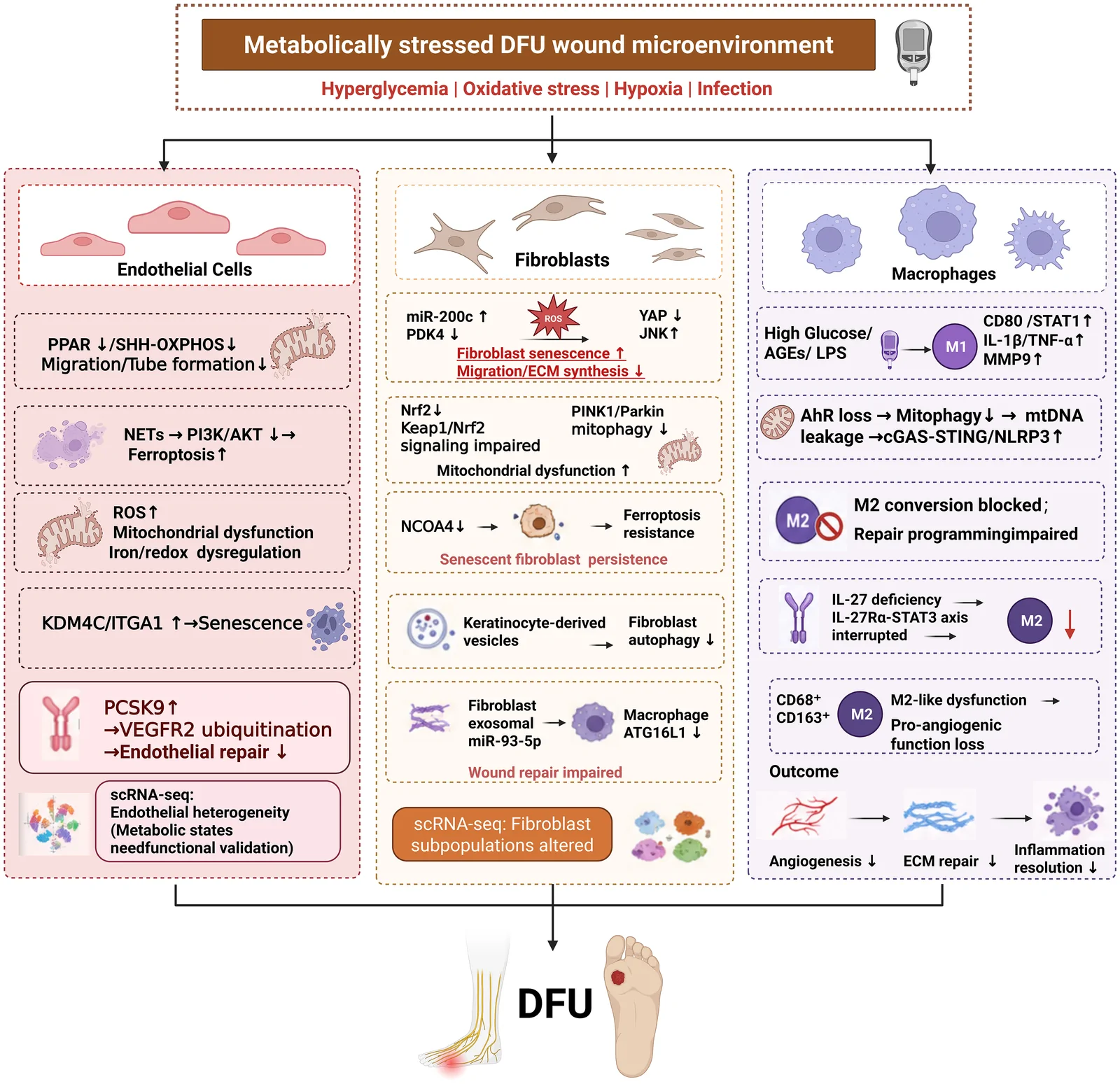
Cell-type-specific metabolic reprogramming in diabetic foot ulcers. In the hyperglycemic, oxidatively stressed, hypoxic, and infected DFU wound microenvironment, endothelial cells, fibroblasts, and macrophages undergo distinct metabolic remodeling, including mitochondrial dysfunction, iron–redox imbalance, ferroptosis, senescence, impaired mitophagy, and non-resolving inflammatory activation. These changes impair angiogenesis, extracellular matrix repair, inflammation resolution, and wound closure. Created with BioRender.com.

DFUs arise in a chronic wound microenvironment shaped by hyperglycemia, oxidative stress, hypoxia, impaired perfusion, and infection. These stresses induce cell-type-specific metabolic remodeling in endothelial cells, fibroblasts, keratinocytes, and immune cells, leading to defective angiogenesis, impaired matrix repair, delayed re-epithelialization, and persistent inflammation. Single-cell studies further support cellular heterogeneity in DFU tissues, including altered epidermal differentiation, endothelial states, fibroblast subsets, and immune-cell phenotypes (121–123) (**Figure 4**).

### 5.1 Endothelial cells

In the DFU microvascular niche, endothelial metabolic remodeling mainly affects mitochondrial energy production, iron–redox balance, and angiogenic repair capacity. Because endothelial cells are required for neovascularization and tissue perfusion, these metabolic defects directly limit wound vascularization. In human DFU tissues and diabetic wound models, impaired PPAR-related signaling is linked to reduced mitochondrial oxidative phosphorylation and defective endothelial migration and tube formation; restoration of PPAR–SHH signaling improves mitochondrial function and angiogenesis, suggesting that loss of mitochondrial bioenergetic support is a central mechanism of endothelial repair failure (124). Immune-derived oxidative injury provides a second metabolic hit. Neutrophil extracellular traps suppress PI3K/AKT signaling and promote endothelial ferroptotic injury, while endothelial cells from non-healing DFUs show increased ROS, mitochondrial dysfunction, and ferroptosis-associated metabolic stress; correction of iron balance and energy metabolism improves wound repair (125, 126). Additional studies indicate that KDM4C/ITGA1-related senescence and PCSK9-mediated VEGFR2 ubiquitination further weaken endothelial regenerative signaling under glycation- and lipid-related stress (127, 128). Together, endothelial dysfunction in DFUs reflects combined mitochondrial energy failure, iron–redox imbalance, and impaired angiogenic signaling, resulting in insufficient neovascularization and delayed wound healing.

### 5.2 Fibroblasts

In the DFU dermal repair compartment, fibroblast metabolic remodeling is characterized by defective glycolytic adaptation, mitochondrial quality-control failure, oxidative stress, and altered iron handling. This is tissue-relevant because fibroblasts provide extracellular matrix deposition, wound contraction, and granulation tissue formation (129). In human DFU fibroblasts and diabetic wound models, increased miR-200c and reduced PDK4 impair glycolytic adaptation and increase oxidative injury; PDK4 deficiency promotes ROS accumulation, YAP inactivation, and JNK activation, thereby driving fibroblast senescence and reducing repair capacity (130, 131). Mitochondrial quality-control failure further reinforces this phenotype: impaired Nrf2/HO-1 or Keap1/Nrf2 signaling and reduced PINK1/Parkin-mediated mitophagy aggravate oxidative stress, mitochondrial dysfunction, and fibroblast senescence in high-glucose and diabetic wound models (132–134). Iron metabolism also shapes the persistence of dysfunctional fibroblasts. In diabetic wound tissue and senescent fibroblast models, NCOA4 downregulation impairs ferritinophagy and increases ferroptosis resistance, allowing senescent fibroblasts to remain in the wound bed (135). In addition, abnormal vesicle-mediated signaling between keratinocytes, fibroblasts, and macrophages disrupts autophagy-related repair programs and further delays healing (136, 137). Overall, fibroblast dysfunction in DFUs reflects impaired glycolytic flexibility, mitochondrial quality-control failure, redox imbalance, and altered iron-dependent cell fate, leading to senescence, weak matrix repair, and poor granulation tissue formation.

### 5.3 Macrophages

In the DFU immune compartment, macrophage immunometabolic remodeling is mainly defined by failure to resolve inflammation and transition toward tissue repair. Because macrophages coordinate pathogen clearance, angiogenesis, matrix remodeling, and repair-cell activation, persistent metabolic stress locks the wound into a chronic inflammatory state (138). In high-glucose/AGE and LPS-exposed macrophage–fibroblast cocultures, macrophages acquire a sustained inflammatory phenotype with increased IL-1β, TNF-α, and MMP9 release, which suppresses fibroblast migration and repair-associated signaling; this suggests that hyperglycemia and infection jointly convert macrophage metabolism into a non-resolving inflammatory program that impairs stromal repair (139). Mitochondrial quality control provides a more direct metabolic mechanism. In diabetic wound models, AhR deficiency suppresses mitophagy, promotes mitochondrial DNA leakage, and activates cGAS–STING–NLRP3 signaling, indicating that defective mitochondrial clearance links metabolic stress to persistent innate immune activation and delayed healing (140). Conversely, IL-27/IL-27Rα–STAT3 signaling supports a repair-associated macrophage response and improves wound closure, suggesting that restoration of reparative immune programming can counteract chronic DFU inflammation (141). Although CD68^+^ CD163^+^ macrophages are detectable in human DFU tissues, marker expression alone does not prove effective repair function (142). Together, macrophage dysfunction in DFUs reflects impaired mitochondrial quality control, persistent innate immune activation, and failure of repair-associated immune remodeling, thereby sustaining inflammation and delaying angiogenesis, matrix repair, and wound closure.

## 6 Diabetic peripheral neuropathy and metabolic reprogramming

**Figure 5:**
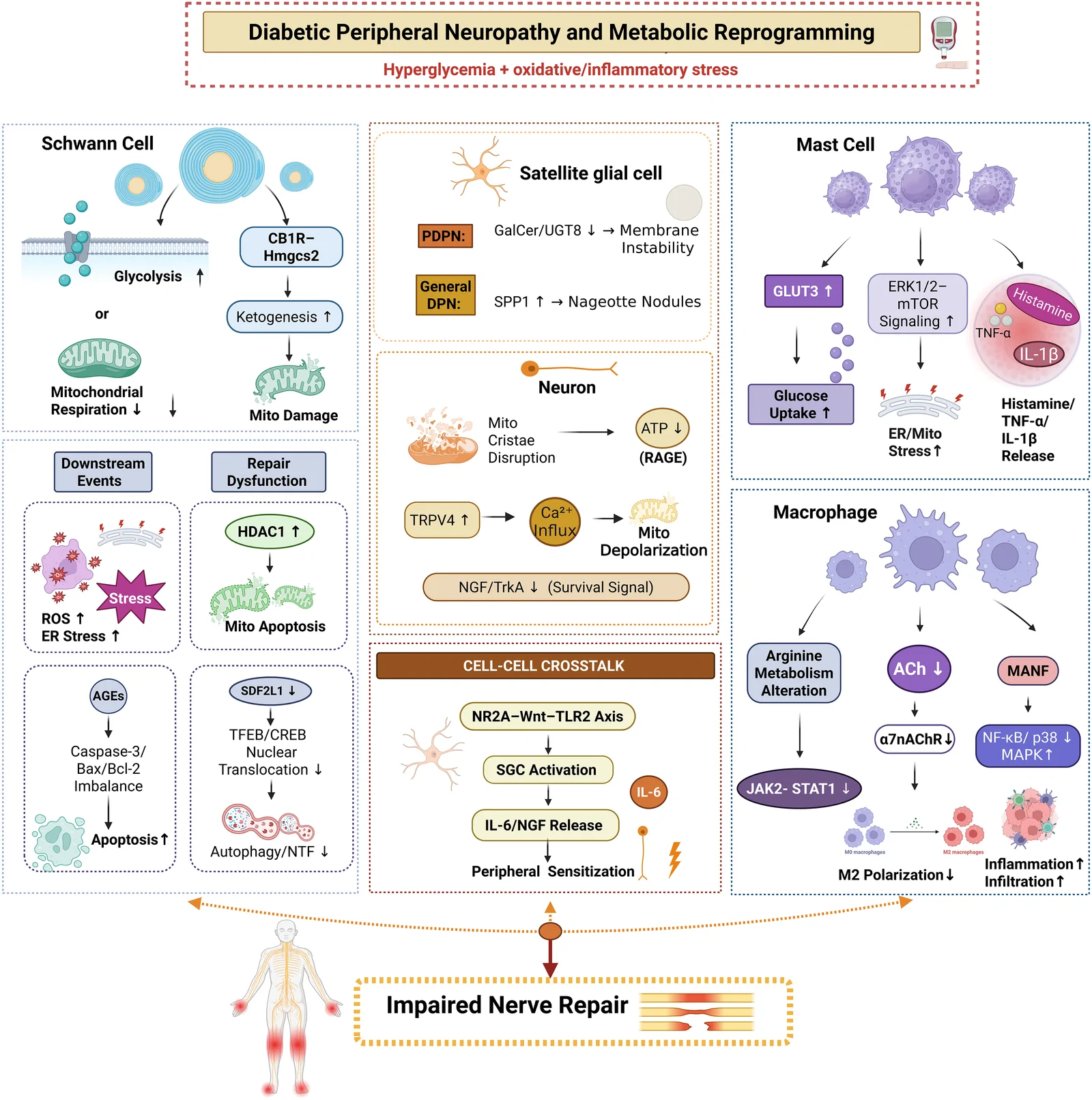
Cell-type-specific metabolic reprogramming in diabetic peripheral neuropathy. Hyperglycemia and oxidative/inflammatory stress reprogram Schwann cells, sensory neurons, satellite glial cells, mast cells, and macrophages within peripheral nerves and dorsal root ganglia. These changes involve mitochondrial dysfunction, altered glucose and lipid handling, calcium–mitochondrial stress, neuron–glia crosstalk, and immune activation, ultimately impairing nerve repair and promoting neuropathic injury. Created with BioRender.com.

DPN arises from metabolic and inflammatory disturbances within peripheral nerves and dorsal root ganglia. Increasing evidence indicates that diabetic stress reshapes the functional states of Schwann cells, sensory neurons, satellite glial cells, and immune cells, thereby disrupting axonal support, neuron–glia communication, and local immune homeostasis (143–155). These cell-type-specific alterations provide the basis for understanding how systemic metabolic disease is translated into peripheral neurodegeneration and neuropathic pain (**Figure 5**).

### 6.1 Schwann cells

Schwann cells normally provide metabolic and structural support to peripheral axons, but under diabetic conditions this supportive phenotype is progressively replaced by a stress-adapted and repair-deficient state. Hyperglycemia alters Schwann-cell bioenergetics and increases oxidative burden, placing mitochondrial function and redox buffering under sustained pressure (143). Within this context, CB1R-driven Hmgcs2 activation and ketone-body production are best interpreted not simply as an isolated ketogenesis pathway, but as evidence of maladaptive substrate reallocation: a compensatory attempt to adjust energy supply that ultimately aggravates mitochondrial injury and energy disequilibrium (145). The same loss of metabolic resilience is reinforced by polyol-pathway activation, glycation-associated stress, defective autophagy, and apoptosis-related signaling, all of which converge on impaired proteostasis and reduced cellular survival (152, 153). Epigenetic and lysosomal-autophagic abnormalities further extend this injury from acute metabolic stress to failed repair, as HDAC1-related mitochondrial apoptosis and SDF2L1-dependent disruption of TFEB/CREB nuclear signaling weaken neurotrophic and regenerative programs (154, 155). Thus, Schwann-cell reprogramming in DPN represents a transition from axon-supportive metabolism to glial metabolic insufficiency, in which abnormal fuel handling, oxidative damage, and defective repair jointly compromise peripheral nerve integrity.

### 6.2 Dorsal root ganglion neurons

In dorsal root ganglion (DRG) sensory neurons, diabetic metabolic stress is translated into neural injury through disruption of calcium–mitochondrial coupling and trophic survival signaling. The pathogenic importance of TRPV4 lies in its position at the interface between altered excitability and mitochondrial failure: excessive Ca^2+^ influx promotes mitochondrial depolarization, oxidative stress, and apoptotic signaling, thereby linking ion-channel dysregulation to both neuronal degeneration and neuropathic pain (146). Impaired NGF/TrkA signaling fits the same disease logic because reduced trophic input limits the ability of injured sensory neurons to maintain survival programs under metabolic stress (147). These findings indicate that neuronal metabolic reprogramming in DPN is not merely a change in energy metabolism, but a broader failure of homeostatic coupling between excitability, mitochondrial function, and survival signaling. This mechanism helps explain how diabetic stress produces both structural sensory-neuron injury and functional pain hypersensitivity.

### 6.3 Satellite glial cells

Satellite glial cells shape the DRG niche by maintaining metabolic and signaling communication around sensory neurons, and diabetic stress appears to convert this regulatory role into a source of local sensitization. Reduced galactosylceramide in painful DPN suggests that sphingolipid remodeling disturbs the membrane and lipid-signaling environment required for stable neuron–glia interaction (148). In this setting, activation of the NR2A–Wnt–TLR2 axis and increased IL-6 and NGF expression should be read as part of a glial inflammatory-metabolic response rather than as a separate inflammatory pathway (150). The relevance of this response is strengthened by human DRG evidence showing Nageotte nodules after sensory-neuron loss, indicating that altered glial–neuronal homeostasis is associated with tissue-level neurodegeneration (149). Therefore, SGC reprogramming in DPN can be summarized as lipid-metabolic remodeling of the DRG microenvironment that lowers the threshold for inflammatory activation and peripheral sensitization.

### 6.4 Mast cells

Mast cells contribute to DPN by converting diabetic metabolic cues into neuroimmune amplification. Changes in GLUT3, ERK, and mTOR-related signaling indicate that these cells undergo altered nutrient-sensing and activation control in the diabetic nerve environment (144). This metabolic shift is functionally consequential because it facilitates degranulation and the release of histamine, tryptase, TNF-α, and IL-1β, which intensify local inflammatory injury and aggravate nerve dysfunction (144). The attenuation of neuropathic progression in mast-cell-deficient diabetic mice further suggests that mast cells are not merely recruited to an already inflamed tissue, but participate in maintaining the inflammatory state that damages peripheral nerves (144). In this sense, mast-cell reprogramming represents a glucose- and mTOR-linked immunometabolic mechanism through which diabetic stress is converted into sustained neuroinflammation.

### 6.5 Macrophages

Macrophage involvement in DPN reflects inflammatory remodeling of the peripheral nerve and DRG microenvironment, with metabolic regulation likely contributing but not yet fully resolved. Increased macrophage accumulation in the DRG and sciatic nerve, together with NF-κB p65 and p38 MAPK activation, indicates that diabetic nerves acquire a pro-inflammatory immune state associated with impaired nerve conduction and reduced intraepidermal nerve fiber density (151). The ability of mesencephalic astrocyte-derived neurotrophic factor (MANF) to reduce macrophage accumulation and suppress these inflammatory pathways supports a functional role for macrophage-driven neuroinflammation in disease progression (151). However, the evidence for specific macrophage metabolic rewiring in DPN remains less direct than that for Schwann-cell or neuronal injury. Findings from broader diabetic complication models, such as arginine deprivation or citrulline supplementation, may point toward redox and amino-acid-linked inflammatory regulation, but they should not be overextended as established DPN macrophage mechanisms without direct supporting evidence (156). Macrophage reprogramming should therefore be framed as an inflammatory-metabolic process that sustains neural injury, while acknowledging that its precise metabolic dependencies in DPN remain incompletely defined.

## 7 Diabetic cardiovascular complications and metabolic reprogramming

**Figure 6:**
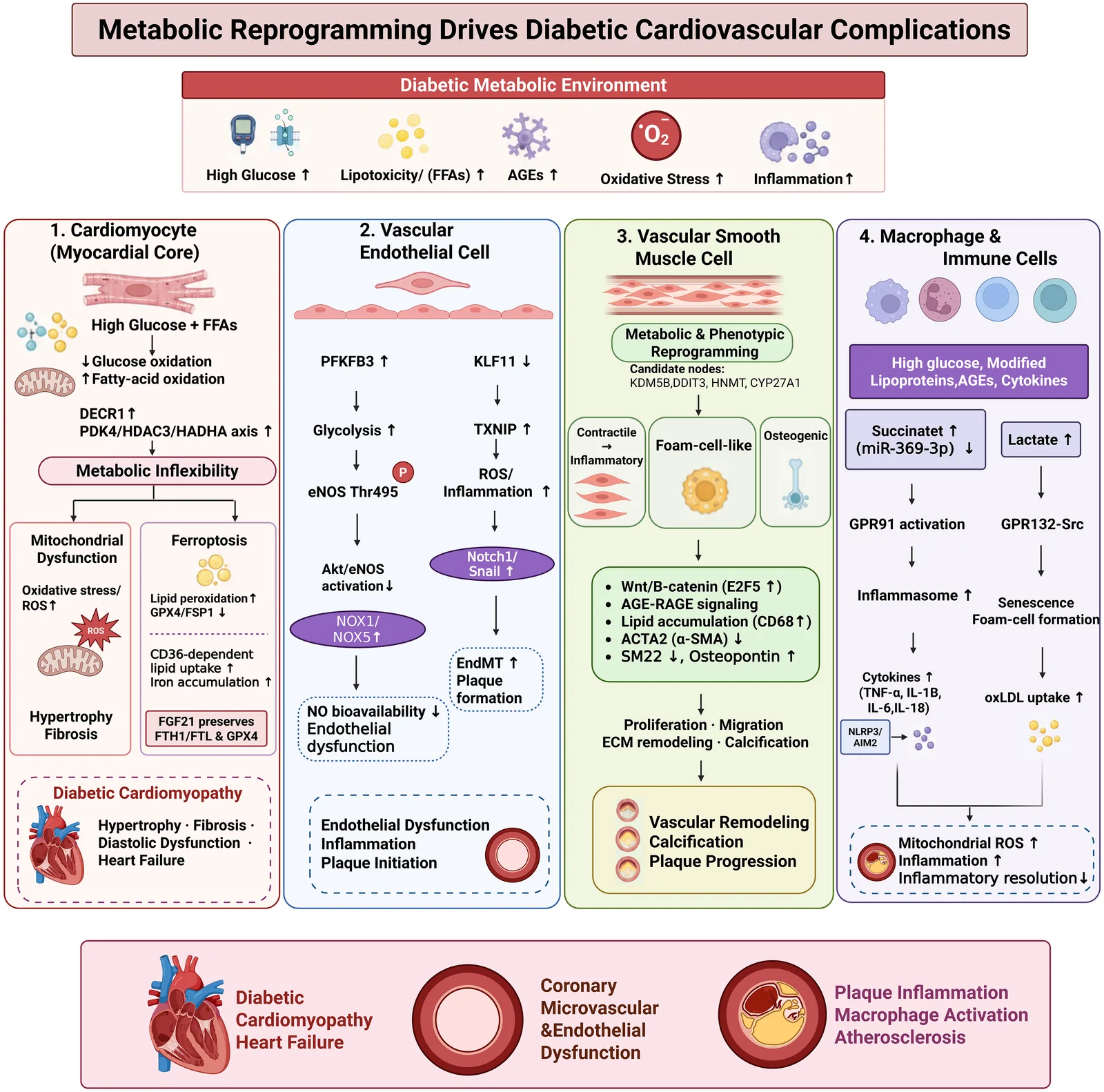
Cell-type-specific metabolic reprogramming in diabetic cardiovascular complications. The diabetic metabolic environment, characterized by high glucose, lipotoxicity, AGEs, oxidative stress, and inflammation, reprograms cardiomyocytes, vascular endothelial cells, vascular smooth muscle cells, and macrophage/immune cells. These changes drive substrate inflexibility, mitochondrial and ferroptotic injury, endothelial dysfunction, VSMC phenotypic switching, foam-cell formation, plaque inflammation, and diabetic cardiomyopathy. Created with BioRender.com.

Diabetic cardiovascular complications are driven in part by metabolic remodeling of myocardial and vascular cells, contributing to diabetic cardiomyopathy and accelerated atherosclerosis (157). These complications involve distinct but interacting programs in cardiomyocytes, endothelial cells, vascular smooth muscle cells, and macrophages, through which diabetic stress disrupts cardiac energetics, vascular homeostasis, plaque stability, and inflammatory resolution (**Figure 6**).

### 7.1 Cardiomyocytes

In cardiomyocytes, diabetic stress produces a state of substrate inflexibility, in which the myocardium becomes increasingly dependent on fatty-acid oxidation while glucose oxidation is suppressed (158). This shift is not merely an adaptive change in fuel preference, because excessive fatty-acid utilization increases mitochondrial workload and promotes oxidative injury. DECR1 upregulation fits this metabolic logic by activating the PDK4/HDAC3/HADHA axis, thereby reinforcing fatty-acid oxidation and linking altered substrate handling to mitochondrial damage, oxidative stress, cardiomyocyte hypertrophy, and fibrosis (159). Ferroptosis further extends cardiomyocyte metabolic injury from energy imbalance to lipid–iron–redox failure. In diabetic hearts, reduced antioxidant defenses and disturbed iron-handling proteins indicate that cardiomyocytes become less able to buffer iron-dependent lipid peroxidation (160). FGF21 preserves FTH1/FTL and GPX4 expression and limits iron accumulation and lipid peroxidation (161), whereas RDH10 loss increases CD36-dependent lipid uptake and weakens GPX4/FSP1-mediated anti-ferroptotic protection (162). Thus, cardiomyocyte reprogramming in diabetic cardiovascular disease can be summarized as a transition from controlled energy metabolism to fatty-acid-driven mitochondrial and ferroptotic injury, which promotes contractile dysfunction and fibrotic remodeling.

### 7.2 Vascular endothelial cells

In vascular endothelial cells, diabetic metabolic reprogramming mainly appears as a maladaptive glycolytic and redox-inflammatory state that compromises endothelial barrier and vasodilatory function. Although endothelial cells physiologically rely on glycolysis, diabetes-associated PFKFB3 upregulation drives excessive glycolytic flux in a setting where redox control and nitric oxide signaling are already impaired (163). This metabolic acceleration becomes pathogenic because it is coupled to increased eNOS Thr495 phosphorylation, NOX1/NOX5 activity, reduced AKT/eNOS activation, and decreased nitric oxide bioavailability, thereby converting altered glucose metabolism into endothelial dysfunction (164). KLF11 deficiency belongs to the same endothelial injury program by amplifying TXNIP-associated oxidative stress and inflammatory activation, while Notch1/Snail-mediated endothelial-to-mesenchymal transition links metabolic inflammation to structural vascular remodeling and plaque formation (165). Therefore, endothelial reprogramming in diabetes is best understood as excessive glycolysis coupled to oxidative and inflammatory signaling, through which endothelial cells lose vasoprotective function and acquire pro-atherogenic properties.

### 7.3 Vascular smooth muscle cells

In vascular smooth muscle cells, diabetic stress promotes a loss of contractile identity and a shift toward inflammatory, foam-cell-like, and osteogenic phenotypes. This process represents metabolic–phenotypic reprogramming rather than a simple proliferative response, because VSMCs in diabetic arteries acquire features normally associated with macrophages, chondroblast-like cells, or calcifying vascular cells. Spatial multiomics of diabetic anterior tibial arteries supports this interpretation by identifying VSMC trajectories toward pro-inflammatory, macrophage-like/foam-cell-like, and fibroblast/chondroblast-like states, with KDM5B, DDIT3, HNMT, and CYP27A1 emerging as candidate regulatory nodes (166). E2F5-driven Wnt/β-catenin activation further links diabetic stimulation to phenotypic switching, as reduced α-SMA and SM22 and increased osteopontin reflect erosion of the contractile program and acquisition of a remodeling phenotype (167). AGE–RAGE signaling reinforces this transition by promoting lipid accumulation and CD68 expression while reducing ACTA2, indicating macrophage-like foam-cell conversion of VSMCs in the diabetic arterial wall (168). Thus, VSMC reprogramming in diabetic vascular disease can be summarized as metabolic and inflammatory loss of lineage stability, through which contractile vascular cells become plaque-remodeling, lipid-loaded, and osteogenic contributors to atherosclerosis.

### 7.4 Macrophages

In diabetic atherosclerotic lesions, macrophages undergo immunometabolic reprogramming that impairs inflammatory resolution and promotes plaque progression. Succinate signaling provides one route by which altered intermediary metabolism is converted into inflammation: reduced miR-369-3p permits succinate accumulation and GPR91-dependent inflammasome activation, linking mitochondrial metabolic stress to cytokine production and plaque inflammation (169). Lactate signaling represents a parallel mechanism in which glycolytic output becomes a pro-atherogenic signal rather than a passive metabolic by-product. Under high-glucose conditions, lactate activates GPR132–Src signaling, promoting macrophage senescence, oxLDL uptake, and foam-cell formation (170). Hematopoietic inflammasome activation through NLRP3 and AIM2 further expands this inflammatory program at the systemic immune level, increasing lesion inflammation and accelerating diabetic atherosclerosis (171). Patient data showing reduced PBMC miR-369-3p or activation of GPR132–Src are consistent with these mechanisms, but they should be interpreted cautiously because they remain associative and are not equivalent to plaque macrophage-specific causal evidence (169, 170). Therefore, macrophage reprogramming in diabetic atherosclerosis is best framed as metabolite-driven inflammatory amplification, in which succinate, lactate, and inflammasome signaling prevent resolution and favor foam-cell accumulation.

## 8 Discussion

In this review, we summarized the shared metabolic reprogramming mechanisms involved in diabetes and its complications, including glucose, lipid, and amino acid metabolic remodeling, mitochondrial dysfunction, immunometabolic changes, and metabolic–epigenetic regulation. We further discussed five major diabetic complications—DKD, DR, DFU, DPN, and diabetic cardiovascular complications—from a cell-type-specific perspective. Although previous reviews have discussed metabolic reprogramming in selected diabetic complications, particularly diabetic kidney disease and diabetes-associated atherosclerosis, few have systematically compared metabolic alterations across multiple diabetic complications or clearly distinguished shared metabolic programs from tissue-specific pathological responses (9, 25, 61, 163). Therefore, this review highlights how shared metabolic stress contributes to organ- and cell-specific diabetic tissue injury.

Diabetic complications share several core features of metabolic reprogramming, including glucose and lipid metabolic reprogramming, mitochondrial dysfunction, ferroptosis-related oxidative stress, immunometabolic remodeling, and metabolic–epigenetic regulation. However, these shared mechanisms differ across organs and cell types, leading to distinct pathological changes in DKD, DR, DFU, DPN, and diabetic cardiovascular complications.

Despite these organ- and cell-specific differences, several upstream metabolic and inflammatory pathways are shared across diabetic complications, including HIF-1α-driven glycolysis, GPX4/SLC7A11-regulated ferroptosis, and TLR4/NF-κB-mediated inflammation. HIF-1α-related glycolytic activation may help cells adapt to high glucose, hypoxia, and inflammatory stress, but persistent activation can promote fibrosis, vascular dysfunction, and tissue injury. Impaired GPX4/SLC7A11 antioxidant defense can increase lipid peroxidation and ferroptosis, especially under hyperglycemic and lipotoxic conditions. At the same time, TLR4/NF-κB-related signaling can drive macrophage, microglial, and glial inflammatory activation. Notably, in different target organs of diabetes, tissue- or cell-specific microenvironments shape divergent pathological outcomes downstream of shared metabolic reprogramming pathways.

Notably, the downstream consequences of these shared pathways are largely determined by tissue-specific microenvironments. For example, although HIF-1α-driven glycolysis is activated in both DKD and DR, the hyperglycemic renal microenvironment favors tubular epithelial cell–macrophage metabolic crosstalk and fibrosis, whereas the hypoxic retinal microenvironment redirects HIF-1 signaling toward Müller-cell-derived VEGF/ANGPTL4 expression and pathological angiogenesis. Likewise, ferroptosis-associated oxidative stress causes endothelial repair failure in DFU but cardiomyocyte injury in diabetic cardiovascular complications because endothelial cells and cardiomyocytes differ substantially in their metabolic demands and functional roles. Similarly, TLR4/NF-κB-mediated inflammatory remodeling promotes macrophage-driven renal fibrosis in DKD but neuroimmune activation and nerve injury in DPN, reflecting differences in immune-cell composition and intercellular metabolic interactions within each tissue. Together, these observations suggest that tissue-specific microenvironments reshape common metabolic programs into distinct pathological responses through different resident cells and intercellular metabolic crosstalk.

Glycolytic stress, ferroptosis-related lipid injury, and immune inflammation may interact with each other and jointly amplify diabetic tissue damage. This convergence suggests that targeting a single pathway may not be sufficient in some diabetic complications, whereas combined or cell-specific strategies may provide a more rational therapeutic direction.

Metabolic stress is closely linked to immune activation and chronic inflammation in diabetic complications. Hyperglycemia, lipotoxicity, oxidative stress, hypoxia, and mitochondrial dysfunction can reprogram macrophages, microglia, endothelial cells, fibroblasts, neurons, and glial cells, thereby promoting inflammatory injury and impaired tissue repair. In DKD, this axis mainly contributes to macrophage-driven renal inflammation and fibrosis (84–89). In DR, it promotes microglial/macrophage activation, vascular leakage, and neurovascular inflammation (91, 92, 111–120). In DFU, it maintains a chronic inflammatory wound environment and delays tissue repair (138–142). In DPN, it contributes to neuroinflammation and neuron–glia dysfunction (143–156). In diabetic cardiovascular complications, it promotes endothelial dysfunction, vascular inflammation, atherosclerosis, and cardiac remodeling (157–171). Overall, this axis links diabetic metabolic stress with organ-specific inflammatory injury. Representative clinical and preclinical evidence, together with approved or emerging therapeutic approaches targeting metabolic reprogramming in diabetic complications, is summarized in **Table 2**.

**Table 2:** Clinical and preclinical evidence and approved or emerging therapeutic approaches targeting metabolic reprogramming in diabetic complications.

Diabetic complication	Study type	Model	Therapeutic approach	Relevant metabolic mechanism	Clinical outcome	References
DKD	Clinical; *in vitro*	Human DKD biopsies; renal cells	Candidate cell-type-specific metabolic stratification	Insulin resistance; glucose/lipid metabolic reprogramming; mitochondrial and immunometabolic remodeling.	Common and cell-type-specific renal injury programs, including filtration-barrier dysfunction and inflammatory/fibrotic alterations.	(60)
DKD	*In vivo*; *in vitro*	DKD mice; endothelial cells	PFKFB3-mediated glycolysis and lactylation targeting	Endothelial glycolysis; lactate/H3K18 lactylation; NLRP3 activation; EndoMT-related fibrosis.	Reduced renal inflammation, endothelial dysfunction, EndoMT and fibrosis in experimental models.	(79, 80)
DKD	*In vivo*; *in vitro*	DKD mice; lymphatic ECs	Empagliflozin/VEGF-C–VEGFR3 pathway modulation	Lymphangiogenesis and lymphatic endothelial-to-mesenchymal transition.	Attenuated renal interstitial fibrosis in experimental DKD.	(83)
DKD	Clinical; *in vivo*; *in vitro*	DKD patients; mice; macrophages	PAD4 inhibition	PAD4/p65/Cmklr1-mediated macrophage recruitment and inflammatory migration.	Reduced macrophage infiltration and tubulointerstitial injury in models.	(87)
DR	Clinical; *in vitro*	Ocular ECs; human PDR/DR samples	Endothelial glycolysis targeting and biomarker/target discovery	Glycolytic reprogramming, angiogenic dysfunction and endothelial mitochondrial heterogeneity.	Pathological angiogenesis, barrier dysfunction and molecular heterogeneity in PDR.	(90, 93)
DR	Clinical; *in vivo*; *in vitro*	Microglia; human PDR hyalocytes; diabetic retina	Microglial/hyalocyte immunometabolic target discovery	ROS-driven microglial activation; macrophage/hyalocyte inflammatory and heme-metabolic remodeling.	Neurovascular inflammation and vitreoretinal remodeling.	(91, 92, 111)
DFU	Clinical	Human DFU tissue	Cell-state and repair-niche mapping	Epidermal differentiation, endothelial heterogeneity, fibroblast subsets and immune microenvironment remodeling.	Pathological wound microenvironment and impaired repair programs.	(121–123)
DFU	Clinical; *in vivo*; *in vitro*	Human DFU tissue; endothelial cells; wounds	PPAR–SHH activation and iron/energy metabolism remodeling	Mitochondrial OXPHOS dysfunction; iron–redox imbalance; ferroptosis-associated injury.	Improved endothelial angiogenesis and wound repair in experimental models.	(124, 126)
DFU	Clinical; *in vivo*; *in vitro*	Human DFU tissue; fibroblasts; macrophages	Fibroblast/macrophage target discovery	Glycolytic adaptation; NCOA4-dependent ferritinophagy/ferroptosis resistance; macrophage phenotype.	Fibroblast senescence, reduced repair capacity and unresolved inflammation.	(130, 131, 135, 142)
DPN	Clinical; *in vivo*	Satellite glial cells; human DRG	Neuro-glial target discovery	Galactosylceramide/sphingolipid remodeling and disruption of neuron–glia homeostasis.	Peripheral sensitization, neuropathic pain and neurodegeneration.	(148, 149)
Diabetic cardiovascular complications	Clinical	Human diabetic failing myocardium	Human-tissue biomarker and candidate-target identification	Ferroptosis-related iron dysregulation, lipid peroxidation, and impaired GPX4-dependent antioxidant defense.	Diabetic failing myocardium showed molecular evidence of ferroptotic injury, cardiac remodeling, and fibrosis; therapeutic efficacy was not evaluated.	(160)
Diabetic cardiovascular complications	Clinical; *in vitro*	Human diabetic arteries; VSMCs	VSMC phenotypic/metabolic target discovery	KDM5B/DDIT3/HNMT/CYP27A1 programs; E2F5–Wnt/β-catenin; AGE–RAGE-mediated foam-cell conversion.	Loss of contractile identity, vascular remodeling and plaque formation.	(166–168)
Diabetic cardiovascular complications	Clinical; *in vivo*; *in vitro*	PBMCs; atherosclerotic plaques; macrophages	Macrophage metabolite and inflammasome targeting	Succinate–GPR91; lactate–GPR132–Src; NLRP3/AIM2 inflammasome activation.	Macrophage senescence, foam-cell accumulation, plaque inflammation and accelerated atherosclerosis.	(169–171)

The relevant metabolic mechanism column corresponds to the major metabolic changes discussed in the manuscript, including glucose/lipid metabolic reprogramming, mitochondrial dysfunction, ferroptosis, immunometabolic remodeling, and epigenetic/metabolic memory. Listed approaches are representative and should not be interpreted as uniformly validated disease-modifying therapies.

Although this review integrates evidence across major diabetic complications, the strength of evidence differs among organs and cell types. Many proposed mechanisms are still based on experimental models, and only a limited number have been validated in human tissues or clinical cohorts. In addition, differences in disease stage, model selection, and analytical methods make direct comparison across complications difficult. Therefore, the metabolic pathways discussed here should be viewed as candidate mechanisms rather than fully established therapeutic targets. Future studies using human samples, longitudinal designs, and cell-specific approaches are needed to clarify their clinical relevance.

## 9 Conclusion

This review provides a comprehensive synthesis of metabolic reprogramming across multiple organs and cell types implicated in diabetic complications, including abnormal glucose and lipid metabolism, mitochondrial dysfunction, ferroptosis, immune activation, and metabolic memory. By comparing cell-type-specific metabolic alterations in the kidney, retina, heart, peripheral nerves, and wound microenvironment, we clarify how shared metabolic reprogramming mechanisms are translated into distinct pathological outcomes within tissue- and cell-specific microenvironmental contexts. These insights deepen the mechanistic understanding of the heterogeneity of diabetic complications and may inform the development of more targeted therapeutic strategies.

Future studies should use single-cell and spatial omics approaches to identify metabolic changes in different cells within diabetic target tissues and clarify how the local microenvironment shapes these changes. Validation of key metabolic pathways in well-characterized clinical cohorts will be essential to determine their prognostic value and therapeutic relevance. From a therapeutic perspective, combination strategies aimed at improving mitochondrial function, correcting lipid metabolic imbalance, inhibiting ferroptosis, and modulating immunometabolism may provide greater benefit than single-pathway interventions. In addition, targeting metabolic memory through epigenetic modulation may help attenuate persistent tissue injury after glycemic control. Ultimately, cell type– and disease stage–specific metabolic therapies guided by molecular and microenvironmental signatures may support more precise prevention and treatment of diabetes-associated complications.
